# A new era in LSD control: Replacing goatpox vaccines with DIVA-enabled homologous strategies in India

**DOI:** 10.1080/21505594.2026.2702710

**Published:** 2026-07-14

**Authors:** Naveen Kumar

**Affiliations:** ICMR-National Institute of Virology, Pune, India

Lumpy skin disease (LSD), a transboundary viral disease of cattle caused by the LSD virus (LSDV), a member of the *Capripoxvirus* genus under the family *Poxviridae*, has emerged as a major threat to the livestock sector in India over the past few years [1]. Since its first report in the country in 2019, LSD has demonstrated rapid geographic expansion, significantly affecting cattle health, productivity, and rural livelihoods. The widespread outbreak in 2022 underscored the vulnerability of the Indian dairy sector, resulting in substantial economic losses estimated at approximately ₹11,589 million [1]. This crisis highlighted the urgent need for effective, safe, and sustainable control strategies, particularly vaccination approaches tailored to the Indian epidemiological landscape.

The *Capripoxvirus* genus, comprising LSD virus (LSDV), sheep pox virus (SPV), and goatpox virus (GPV), exhibits a high degree of genetic similarity and antigenic cross-reactivity [2]. Owing to this relatedness, SPV- or GPV-based vaccines have historically been employed to provide cross-protection against LSD in cattle, particularly in situations where LSDV-specific vaccines were unavailable [3–5].

However, substantial evidence indicates that homologous live-attenuated LSD vaccines confer robust and complete protection against diverse LSDV field strains, whereas heterologous GPV/SPV vaccines typically provide only partial and often inconsistent protection [6–9]. Furthermore, heterologous vaccines are generally administered at significantly higher doses, often up to ten times those of homologous vaccines, to achieve suboptimal protective efficacy [10,11]. Recognizing these limitations, the World Organisation for Animal Health (WOAH) recommends the use of homologous live-attenuated LSDV vaccines for effective disease control and discourage reliance on heterologous goatpox or sheeppox vaccines in cattle, except under emergency circumstances [12]. Even in such situations, WOAH emphasizes the need for independent validation of safety and efficacy of a particular heterologous vaccine strain, prior to large-scale deployment.

In the Indian context, following the severe LSD outbreak in 2022, the Department of Animal Husbandry and Dairying (DAHD), Govt. of India authorized mass vaccination of cattle using a heterologous goatpox virus vaccine (Uttarkashi strain) [13]. This decision was guided by the established concept of cross-protection among capripoxviruses, although the safety and efficacy of this vaccine in cattle had not been validated at the time [1].

Although a temporary decline in LSD incidence was observed during 2023–2024, widespread outbreaks reemerged by mid-2025 despite continued large-scale vaccination campaigns using the goatpox vaccine [14]. This resurgence highlights the persistent vulnerability of the cattle population and raises critical concerns regarding the real-world effectiveness of heterologous vaccination strategies under Indian field conditions.

However, field experience over subsequent years revealed that the Uttarkashi strain-based heterologous goatpox vaccine provides suboptimal protection. Reports of breakthrough infections, inconsistent immune responses, and incomplete prevention of clinical disease highlighted the limitations of this approach, particularly in settings of high transmission pressure [14]. Field investigations in October 2025 across 18 farms in Rajasthan (8,930 cattle) showed low effectiveness (21.7%) of the Uttarkashi goatpox vaccine, with similar LSD incidence in vaccinated (11.2%) and unvaccinated (14.3%) animals [15]. Older cattle (>3 years) were largely unaffected, while younger animals showed similar infection rates irrespective of vaccination, suggesting that the decline in outbreaks from 2022–2023 in adult cattle was primarily due to residual natural herd immunity from the 2022 outbreak rather than annual mass vaccination with the heterologous goatpox vaccine [15]. Immunogenicity studies confirmed weak antibody and cellular responses with goatpox vaccine, unlike strong responses with LSD-specific vaccine. Overall, the goatpox vaccine offers limited protection, supporting a shift to homologous LSD vaccination [15]. These observations reinforced the need for a more effective, homologous vaccination strategy.

In 2022, India developed an indigenous live-attenuated homologous LSD vaccine, derived from a native virus strain (LSDV/India/2019/Ranchi) [16]. The vaccine underwent comprehensive experimental and field evaluations in cattle, demonstrating strong immunogenicity and high safety and efficacy profile [16]. It subsequently received regulatory approval from the Central Drugs Standard Control Organization (CDSCO), Govt of India in 2024 and is now commercially available as Biolumpivaxin® [1]. A defining and globally significant feature of this homologous vaccine is its incorporation of the DIVA (Differentiating Infected from Vaccinated Animals) concept, making it the world’s first DIVA marker vaccine for LSD [17,18]. This unique capability enables precise serological differentiation between naturally infected and vaccinated animals, thereby facilitating robust surveillance, outbreak investigation, and disease control programs. In addition to its DIVA capability, the Ranchi strain-based vaccine has demonstrated an outstanding safety profile under extensive field use. Millions of animals have been vaccinated without any observable local or systemic adverse reactions [1,16]. This is in stark contrast to several globally used live-attenuated LSD vaccines, where the so-called “Neethling response” characterized by localized skin lesions, occasional generalized micronodules, transient fever, and temporary reduction in milk yield is observed in 10–15% of vaccinated animals. The absence of these adverse effects in the indigenous vaccine significantly enhances its acceptability among farmers and reduces post-vaccination economic losses.

In light of compelling scientific evidence supporting the superiority of the homologous vaccine, the Government of India has taken a decisive and forward-looking policy step. In March 2026, the DAHD issued an advisory to phase out the use of the goatpox vaccine for LSD control and transition entirely to the indigenously developed homologous LSD vaccine [19]. This decision reflects a science-driven, evidence-based approach aligned with global best practices that prioritize homologous vaccination for optimal disease control. While the policy decision to phase out the goat pox vaccine is scientifically sound, its successful execution hinges on robust field-level awareness to ensure a seamless transition.

In conclusion, phasing out the goatpox vaccine and adopting a homologous LSD vaccine marks a major advance in India’s animal health strategy, reflecting evidence-based policy and indigenous innovation. The introduction of the world’s first DIVA-enabled LSD vaccine positions India at the forefront of global LSD control, with significant benefits for livestock productivity, dairy resilience, and animal health security.

## Data Availability

No primary data (figures and tables) are included in this article.
